# The past and the future of Alzheimer's disease CSF biomarkers—a journey toward validated biochemical tests covering the whole spectrum of molecular events

**DOI:** 10.3389/fnins.2015.00345

**Published:** 2015-09-29

**Authors:** Kaj Blennow, Henrik Zetterberg

**Affiliations:** Clinical Neurochemistry Laboratory, Department of Psychiatry and Neurochemistry, Institute of Neuroscience and Physiology, The Sahlgrenska Academy at University of GothenburgMölndal, Sweden

**Keywords:** Alzheimer disease, biomarker, cerebrospinal fluid, neurogranin, oligomers, synaptic proteins, tau proteins

## Abstract

This paper gives a short review on cerebrospinal fluid (CSF) biomarkers for Alzheimer's disease (AD), from early developments to high-precision validated assays on fully automated lab analyzers. We also discuss developments on novel biomarkers, such as synaptic proteins and Aβ oligomers. Our vision for the future is that assaying a set of biomarkers in a single CSF tube can monitor the whole spectrum of AD molecular pathogenic events. CSF biomarkers will have a central position not only for clinical diagnosis, but also for the understanding of the sequence of molecular events in the pathogenic process underlying AD and as tools to monitor the effects of novel drug candidates targeting these different mechanisms.

Laboratory medicine tests influence up to 70% of clinical decisions and thus have a central position in clinical medicine (Beastall and Watson, 2013). Biochemical markers for chronic neurodegenerative disorders are especially important, since the slow progression and diffuse symptomatology results in diagnostic difficulties, and tissue sampling with direct visualization of central nervous system (CNS) pathology is not clinically applicable. For this reason, the Alzheimer's disease (AD) arena is in the good situation that a set of highly validated and specific biomarkers are at hand; in addition to amyloid positron emission tomography (PET) and magnetic resonance imaging (MRI) measurements, a set of cerebrospinal fluid (CSF) tests reflecting key aspects of disease pathology are available. This paper comments on some caveats on the road to develop and validate these CSF biomarkers and some recent developments on novel biochemical tests.

## Early assay developments

The story on modern AD biomarker development started in 1995 with a series of publications on enzyme-linked immunosorbent assays (ELISA) based on monoclonal antibodies to measure CSF levels of total tau (T-tau) and phosphorylated tau (P-tau) and the 42 amino acid isoform (Aβ42) of β-amyloid (Blennow et al., 1995; Motter et al., 1995). These papers reported a marked increase in CSF T-tau and P-tau accompanied by a marked decrease in Aβ42 in AD (Blennow et al., 1995; Motter et al., 1995). The following years, many research reports consistently showed that the “AD profile” of increased CSF levels of T-tau and P-tau together with decreased Aβ42 had high sensitivity and specificity, both in the range of 85–90%, to identify AD dementia, for review see (Blennow and Hampel, 2003). Since these three CSF biomarkers reflect key elements of AD pathophysiology, i.e., neuronal degeneration (T-tau), tau pathology (P-tau), and amyloid plaques (Aβ42), they are often termed the “core” AD biomarkers (Hampel et al., 2004).

## The problem with studies based on clinical diagnosis

The vast majority of studies were cross-sectional and the diagnoses were based on the exclusion criteria published in 1984 by the National Institute of Neurological and Communicative Disorders and Stroke and the Alzheimer's Disease and Related Disorders Association (NINCDS-ADRDA). In the studies evaluating the diagnostic performance of CSF biomarkers, the diagnostic entity “probable AD” based on the NINCDS-ADRDA criteria, i.e., an exclusion diagnosis made on pure clinical grounds, was used as gold standard in the evaluation of the CSF biomarkers (McKhann et al., 1984). For logical reasons, the poor diagnostic accuracy of these criteria (Knopman et al., 2001), and the overlap in pathology between AD and other dementias, such as Lewy body dementia and vascular dementia (Blennow et al., 2006), made it impossible to achieve full diagnostic separation between AD and aging or other dementias using biomarkers.

## The issue of biomarker-positive elderly

The introduction of amyloid PET in the arsenal of AD biomarkers marked a major change in AD biomarker research, since it became clear that 20–30% of apparently healthy elderly showed positive on scans (Klunk, 2011). In 2006, the first study showed that high amyloid ligand retention on amyloid PET almost completely corresponds to low CSF Aβ42 (Fagan et al., 2006), and vice versa, a finding that has been verified in numerous subsequent studies, for review see Blennow et al. (2015). This knowledge rather quickly changed the view on how to interpret low CSF Aβ42 levels in cognitively intact elderly, from poor assay quality or biomarker performance to an indicator of preclinical AD.

In support of this, reliable biomarkers for cerebral β-amyloidosis also made it possible to follow cognitively normal Aβ-positive individuals over time. Such longitudinal studies are relevant given the fact that many individuals with AD neuropathology could be dementia-free when they died. Longitudinal Aβ biomarker studies suggest that the majority of Aβ-positive individuals followed over many years develop cognitive impairment and eventually dementia. In other words, if the dementia-free individuals with AD neuropathology would have lived 5–10 years longer they would most likely have developed AD (Buchhave et al., 2012).

## Turning direction toward early diagnosis

The failures of Phase 2 and 3 trials testing anti-Aβ disease-modifying drug candidates on AD patients in the dementia stage initiated a discussion on the whether this type of treatment need to be initiated before the dementia phase of the disease, i.e., before the neurodegenerative process is too severe and widespread (Blennow, 2010). An attractive option was therefore to perform further trails on AD patients in the mild cognitive impairment (MCI) stage of the disease. However, this would also introduce diagnostic challenges since MCI is a heterogeneous syndrome that may have many different underlying causes. Around 50–60% of MCI cases have prodromal AD (Dubois et al., 2007), meaning that they have underlying AD pathology and will progress to AD with dementia. MCI symptoms may also be caused by other neurodegenerative disorders such as Lewy body dementia and vascular dementia or be due to age-related benign cognitive disturbances, stress and depression. Further, symptoms in MCI cases are by definition vague and diffuse, which makes it impossible to diagnose AD clinically in unselected MCI cohorts (Petersen et al., 1999). This created a need to test if the CSF biomarkers have value also for early diagnosis.

In 1999, a first paper showed that MCI patients progressing to AD with dementia, which is sometimes called “converting,” during the clinical follow-up period had the typical AD CSF profile of high T-tau and P-tau together with low Aβ42, and levels were equally abnormal in the MCI and the dementia stage in cases with longitudinal sampling (Andreasen et al., 1999). In the first studies, no MCI group with long clinical follow-up, which is needed to ascertain that stable MCI cases will not progress, was presented. The first study with an extended clinical follow-up period, showed that the AD CSF profile had a 95% sensitivity for prodromal AD at a specificity of 83–92% against controls and stable MCI cases and MCI cases that proved to have other dementias (Hansson et al., 2006). A series of large multi-center studies could verify such a high diagnostic accuracy of the AD CSF biomarker profile to identify prodromal AD (Mattsson et al., 2009; Shaw et al., 2009; Visser et al., 2009).

## Entering diagnostic criteria

In 2007, the International Work Group (IWG) published the first research criteria for the diagnosis of prodromal AD for New Research Criteria for the Diagnosis of AD (Dubois et al., 2007). These criteria provided a new conceptual framework stating that AD could be diagnosed based on the combination of a clinical phenotype of episodic memory disturbances and one or more abnormal AD biomarker including CSF biomarkers (Aβ and tau proteins), volumetric MRI and amyloid PET) (Dubois et al., 2007). In 2011, similar, but not identical, criteria for MCI due to AD (Albert et al., 2011) and dementia due to AD (McKhann et al., 2011) were published by the National Institute on Aging—Alzheimer's Association (NIA-AA) workgroups on diagnostic guidelines for AD. The IWG criteria for prodromal AD and NIA–AA criteria for MCI due to AD are similar, and most cases fulfilling one set of criteria will also fulfill the other, but the NIA–AA criteria allow for assessment of the likelihood of being correctly diagnosed, with both amyloid and (neuronal) injury biomarker positive cases having the highest likelihood (Visser et al., 2012). In the updated IWG-2 criteria (Dubois et al., 2014), CSF biomarkers got a more central role, together with amyloid PET, due to their high diagnostic performance (Hansson et al., 2006; Li et al., 2007; Brys et al., 2009; Snider et al., 2009; van Rossum et al., 2012), while downstream topographical AD biomarkers, such as volumetric MRI and FDG- PET, were judged to function better in monitoring disease course in AD.

## Cut-offs and clinical interpretation

The issue of identifying unified cut-offs for the CSF biomarkers was brought up in the updated IWG-2 criteria (Dubois et al., 2014). For CSF biomarkers, this problem stems from differences in pre-analytical procedures between clinics and in analytical procedures between laboratories, and not the least from variability in manufacturing procedures for the assays, with batch-to-batch variations (Mattsson et al., 2013). To overcome these problems, several standardization initiatives have been launched with the aim to minimize this type of variability, including the Global Biomarker Standardization Consortium (GBSC) and the International Federation of Clinical Chemistry and Laboratory Medicine (IFCC) Work Group for CSF proteins, that aims to develop certified reference materials and methods to serve as “gold standards” for CSF biomarker measurements (Carrillo et al., 2013). These initiatives will, together with novel validated assays produced under rigorous quality control measures and CSF biomarker methods run on fully automated lab analyzers, allow to uniform cut-off levels for diagnosis, and a more widespread use of CSF biomarkers in the routine clinical diagnostic setting.

However, for common age-related disorders such as diabetes type II and hypertension, there is no distinct line between health and disease, and recommended cut-offs must therefore be based on estimations of risk and values in the individual patient must always undergo clinical interpretation. The situation is the same for AD, with an increasing overlap in neuropathological changes (Mountjoy et al., 1983; Mann et al., 1984; Hansen et al., 1988) and in CSF biomarker levels (Andreasen et al., 1999a; Mattsson et al., 2012) between aging and AD with increasing age. Indeed, studies comparing the diagnostic performance of CSF biomarker levels (Aβ42) and amyloid PET show that the overlap around the proposed cut-off for both biomarker modalities (Mattsson et al., 2014) makes it questionable to dichotomize results into biomarker (CSF Aβ42 or amyloid PET) “positive” or “negative.” The tradition in Laboratory medicine is to report the actual concentration of a biomarker back to the clinician who based on clinical experience interprets biomarker values near the cut-off with caution.

Ratios such as T-tau/Aβ42, combining one injury and one amyloid biomarker, are commonly evaluated in clinical biomarker studies, and often found to perform better than either biomarker alone. Even if this type of ratios show excellent diagnostic separation in selected AD and control populations, they may be difficult to implement in unselected populations in the clinic. This is since an increase in CSF T-tau in patients with minor stroke, encephalitis or CJD will have a very high ratio despite having normal CSF Aβ42, and thus no indication of amyloid pathology (Blennow et al., 2006).

## The putative *APOE* dependence of CSF Aβ42

The apolipoprotein E (*APOE*) ε4 allele is the main genetic risk factor for AD (Bertram and Tanzi, 2008). In the late 1990ies, several studies reported that AD patients possessing the *APOE* ε4 allele had lower CSF Aβ42 than those without this gene variant (Galasko et al., 1998; Hulstaert et al., 1999). This association is present also in cognitively normal elderly (Prince et al., 2004). In contrast, CSF tau levels do not depend on the ε4 allele (Andreasen et al., 1999b).

These results raised the question whether the ApoE4 isoform modulates brain and CSF Aβ levels through a physiological mechanism. Some studies on mice found that the ApoE isoforms differentially regulates Aβ clearance, and suggested that the *APOE* genotype contribute to AD risk by differentially regulating clearance of Aβ the brain throughout life (Castellano et al., 2011; Verghese et al., 2013). In a clinical study challenging this hypothesis, MCI patients stratified by for cortical amyloid deposition as evaluated by amyloid PET, amyloid positive cases had low CSF Aβ42 levels, and amyloid negative cases normal Aβ42 levels, independently of ε4 status (Lautner et al., 2014). These findings indicate that the gene-dose dependent association between the *APOE* ε4 allele and Aβ42 is caused by more severe amyloid deposition in patients that are ε4 carriers. In support of this conclusion, there is no association between CSF Aβ42 and the *APOE* ε4 allele in young individuals, that are likely to be free of brain amyloid deposition (Lautner et al., 2014), and thus no evidence of a physiological effect on Aβ clearance in man. In addition, these findings show that there is no need for *APOE* allele-dependent cut-off levels for CSF Aβ42.

## Compensating for differences in basic Aβ production—the Aβ42/Aβ42 ratio

Except for Aβ42, the CSF contains several other Aβ isoforms, the most abundant variant being Aβ40 (Portelius et al., 2006). Even if CSF Aβ40 is relatively unchanged in AD, the CSF Aβ42/Aβ40 has been suggested to have stronger diagnostic accuracy for AD compared to CSF Aβ42 alone (Hansson et al., 2007). The explanation may be that the ratio normalizes individuals according to their Aβ production level, so that low CSF Aβ42 can be more easily detected in “high Aβ producers” and vice versa (Lewczuk et al., 2015). Recent studies show that the CSF Aβ42/Aβ40 ratio is valuable also in the clinical setting (Dumurgier et al., 2015).

## The everlasting promise of blood biomarkers for AD

The CSF is continuous with the brain extracellular space, with a free exchange of molecules that makes it possible to monitor brain biochemistry by CSF analyses. Nevertheless, since blood is more accessible than CSF, for which a lumbar puncture is needed, blood biomarkers are desirable both for clinical diagnosis or screening and for multiple sampling in clinical trials. However, there are several circumstances that make blood a more challenging matrix than CSF for brain biomarkers. First, peripheral blood (plasma and serum) and the brain are separated by the blood-brain barrier, making only a small fraction of brain proteins enter the bloodstream. Second, the minute amounts of brain proteins entering the blood will be diluted in a compartment containing very high levels of other proteins such as albumin and IgG, introducing a high risk of interference in analytical methods (Blennow and Zetterberg, 2015). Third, brain proteins in the bloodstream will be subjected to degradation by proteases, degradation in the liver or clearance in the kidneys, which will introduce a risk of confounding data. As an example, the Australian Imaging Biomarkers and Lifestyle (AIBL) research team have reported that plasma Aβ levels are influenced by inflammatory and renal function covariates and that absolute levels of either Aβ40 or Aβ42 do not associate with AD or neocortical Aβ burden (Rembach et al., 2014). These factors make development of blood biomarkers for chronic neurodegenerative disorders challenging and limits the potential of blood samples as biomarker sources for AD.

One possible approach is to apply hypothesis-free proteomics, lipidomics, and similar methods in the search for AD blood biomarkers. Such studies report combinations of proteins, lipids, metabolites, or other molecules that discriminate AD from controls, and propose such panels as novel AD blood biomarkers, for review see (Henriksen et al., 2014). These studies often screen a high number of unselected molecules, each showing a marked overlap between AD and controls. However, when combining a number of molecules using multivariate statistics, a diagnostic separation is found. This type of studies have several challenges. First, analytical standardization is difficult for a panel of analyses consisting of high number of proteins or molecules with different characteristics (O'Bryant et al., 2015). Second, pre-analytical factors, such as influence of age, gender, other diseases, medications, food-intake, or physical activity may vary considerably between these molecules, or are not known or not examined. Third, patient and control cohort differences may influence outcome, but the panel is often evaluated in a “training” and “validation” set of patients and controls from the same cohort. Last, but not least, the issue of potential statistical over-fitting of data to identify a “biomarker panel” from a very large number of molecules in samples from a specific cohort with limited number of cases may introduce bias. For these reasons, such panels of molecules unrelated to AD pathogenesis often fail to replicate in independent clinical cohorts (Zhao et al., 2015), or alternative protein biomarker panels are proposed in the different studies (Henriksen et al., 2014).

## Biochemical tests covering the whole spectrum of molecular events

Despite that the core CSF AD biomarkers reflect central pathogenic mechanisms of the disease, novel biomarkers to monitor additional important molecular mechanisms in AD are constantly sought. Two important aspects of AD pathophysiology are soluble oligomeric Aβ species and synaptic dysfunction and degeneration.

### Oligomeric Aβ may give clues to disease pathogenesis

Amyloid plaques are composed of aggregated Aβ, but research during the last decade has put focus on soluble oligomers of Aβ that may inhibit long-term potentiation (LTP) and cause tau hyperphosphorylation and neuritic dystrophy (Walsh et al., 2002; Jin et al., 2011), possibly by specifically affecting synapses and disturbing synaptic signaling pathways (Pozueta et al., 2013). LTP is thought to be the key mechanism behind memory encoding, the possible causation between Aβ oligomers and synaptic dysfunction and damage has evolved into an active area of research. However, LTP cannot be measured *in vivo* in man, and a key question is whether there is a primary Aβ oligomer-induced deficit in LTP in the early stages of AD, or whether the synaptic degeneration in AD causes memory impairment through other mechanisms, with LTP deficits being downstream consequences of the synaptic dysfunction and loss. Tools to study these molecular mechanisms in man would thus be valuable.

Aβ oligomers, ranging from dimers, trimers, dodecamers, and larger molecular weight species have been found to be present in CSF (Klyubin et al., 2008; Handoko et al., 2013). However, in addition to the molecular heterogeneity, CSF Aβ oligomer levels are very low, making reliable quantification challenging. Indeed, different studies have applied a wide variety of methodologies to allow quantification of these soluble aggregates, such as fluorescence correlation spectroscopy (Pitschke et al., 1998), bio-barcode assay (Georganopoulou et al., 2005), misfolded protein assay (Gao et al., 2010), ELISA with the same monoclonal antibody both for capture and detection (Fukumoto et al., 2010), flow cytometry based assays (Santos et al., 2012), immunoprecipitation and Western blot (Handoko et al., 2013), and ultrasensitive bead-based immunoassays (Savage et al., 2014). Several studies have found increased Aβ oligomer levels in CSF of AD patients (Pitschke et al., 1998; Georganopoulou et al., 2005; Fukumoto et al., 2010; Gao et al., 2010; Handoko et al., 2013; Holtta et al., 2013; Savage et al., 2014), but with large overlap with control groups, while other studies have reported no change (Santos et al., 2012; Bruggink et al., 2013; Jongbloed et al., 2015) or lower levels (Sancesario et al., 2012).

The reason for these contradictory results is unclear, but may include analytical shortcomings, variability in how and in which type of oligomer assemblies are secreted from the brain to the CSF, instability of Aβ oligomers in CSF or during the analytical procedures, or other factors. Nevertheless, if these analytical shortcomings and variability between studies can be overcome, CSF Aβ oligomers measurements may provide important clues to disease pathogenesis when applied in longitudinal studies in the different stages of AD and related to both neuropsychological evaluations and other AD biomarkers such as amyloid PET and MRI measurements. However, the finding in several studies that CSF Aβ oligomer levels correlate with disease severity, with higher CSF levels in more advanced disease (Fukumoto et al., 2010; Santos et al., 2012; Savage et al., 2014), does not support that they are associated with early disease pathogenesis.

### Synaptic biomarkers enter the arena

Synapses are the building blocks of neuronal networks. Synapses consist of a pre-synaptic unit with synaptic vesicles containing the neurotransmitters that upon release, regulated by a delicate machinery of pre-synaptic proteins, bind to post-synaptic receptors at the dendritic spines and activate a cascade of molecular events to advance the signal (Jahn and Fasshauer, 2012). Synaptic dysfunction and degeneration is likely the direct cause of the cognitive deterioration in AD. Synaptic degeneration is an early pathogenic event in AD (Masliah et al., 2001; Scheff et al., 2007), with synaptic loss being more tightly correlated with cognitive impairment than either plaque or tangle pathology (DeKosky and Scheff, 1990; Blennow et al., 1996; Sze et al., 1997). Thus, synaptic biomarkers may serve as a tool to study the link between the molecular pathology and cognitive symptoms.

As mentioned above, there is no method to measure LTP in man, but some synaptic proteins such as neurogranin has been shown to play a critical role in LTP (Wu et al., 2002; Huang et al., 2004). Neurogranin is highly concentrated in dendritic spines, and neurogranin levels are markedly reduced in the hippocampus and the frontal cortex in AD, indicating loss of post-synaptic elements (Davidsson and Blennow, 1998; Reddy et al., 2005). A pilot study using immunoprecipitation and Western blot showed increased CSF levels of neurogranin in AD (Thorsell et al., 2010). The first study using a quantitative immunoassay showed a marked increase in CSF neurogranin in AD dementia and high levels predicted progression to AD dementia among MCI patients (Kvartsberg et al., 2014). Further, in amyloid positive MCI cases, high neurogranin correlated with a more rapid cognitive deterioration during clinical follow-up (Kvartsberg et al., 2014). Among proteins specific for the pre-synaptic part of the synapse, SNAP-25 CSF levels are clearly elevated in AD, also in the prodromal phase of the disease (Brinkmalm et al., 2014a), probably reflecting the ongoing destruction of presynaptic terminals (Davidsson and Blennow, 1998; Brinkmalm et al., 2014b).

## Concluding remarks

Three CSF biomarkers reflecting the core pathological features of AD are available: T-tau (neurodegeneration), P-tau (tau hyperphosphorylation and, potentially, tangle formation), and Aβ42 (plaque pathology). According to revised clinical criteria, these markers may help diagnose AD more accurately and open up the possibility of detecting pre-dementia stages of the disease. At present, their most obvious utility is in clinical trials of novel disease-modifying treatments against AD. In the future, they may help selecting the right treatment for individual patients by making it possible to assess which molecular pathology is most likely to cause the patient's symptom at different stages of the disease. Standardization efforts are now moving the CSF tau and Aβ biomarker tests toward automated clinical-grade assays, which hopefully will become as established and standardized as clinical chemistry tests for other common human diseases. In addition, there is considerable promise that CSF biomarkers will provide *in vivo* measurement of a range of additional pathophysiological processes in AD. New biomarkers including synaptic proteins and Aβ oligomers, will broaden the arsenal toward a panel that covers the whole spectrum of molecular events in AD. The application of such panels in longitudinal clinical studies will give essential additional information of the evolution of pathogenic processes in AD.

### Conflict of interest statement

The authors declare that the research was conducted in the absence of any commercial or financial relationships that could be construed as a potential conflict of interest.
